# On Dislocations of the Thumb at the Metacarpo-Phalangeal Joint, Showing That the Difficulty of Reducing Arises from Malposition of the Tendon of the Long Flexor

**Published:** 1864-12

**Authors:** J. C. Wordsworth


*On Dislocations of the Thumb at the Metacarpo-Phalangeal Joint, showing that the difficulty of reducing arises from Malposition of the Tendon of the Long Flexor.* By J. C. WORDSWORTH, Esq., F. R. C. S.

Many years ago my attention was particularly directed to the subject of dislocations of the thumb and fingers, by a case that occurred in the practice of the London Hospital while I was the house-surgeon. The cause of difficulty in reducing them was so plainly illustrated in this case that I was induced to publish a short notice of it in the pages of the Lancet, together with two other cases that seemed to confirm my conclusions. It had been previously stated, by various writers, that the strong lateral ligaments were the obstacles to reduction; and by other surgeons the difficulty was attributed to malposition of the tendons. Amongst the latter were the late Mr. Stanley in our own country, and Messrs. Lisfranc and Dupuytren in France. At the time I wrote I was not aware that such opinions had been published; I there-gladly avail myself of this opportunity of disclaiming any credit for my share in the elucidation of this important subject, and am content to attempt to illustrate and confirm their opinions.

The case to which I refer was one of *compound* dislocation of the the first phalanx of the thumb upon the dorsum of its metacarpal bone, in which the tendon of the long flexor was found between the ends of the bones, and thus accounted for the difficulty in restoring them to their proper positions. I will then briefly reproduce the essentials of these three cases: for though I have had many opportunities, both in public and private, of confirming this view of the subject, and of testing the practical value of it by reducing dislocations which have baffled others, yet I could not cite any that afford more satisfactory data for the solution of the question.

I gladly embrace this occasion to offer my sincere thanks to many friends, who, being aware of my interest in these cases, have afforded me numerous opportunities of seeing them; and I believe I am at liberty to state they fully approve and confirm what I have to offer on this subject.

Case 1.—A *compound* dislocation of the first phalanx of the thumb, produced by a fall on the extended hand, the phalanx being on the dorsum of the metacarpal bone. A wound extended across, and opened the joint on its palmar aspect. An attempt at reduction was made in the usual manner, simply by extension, and failed. A close scrutiny of the wound showed the tendon of the long flexor between the ends of the bones, having passed round the *ulnar* side of the end of the metacarpal bone, and by traction been drawn across the joint. Various attempts were made to remove the tendon from its new position without doing further mischief; but these being unsuccessful, it was divided with a bistoury. Reduction was at once accomplished, and no displacement recurred.

This case, then, sufficed to convince me of the presence of the obstacle as well as of it sufficiency, affording as it did positive proof that the tendon prevented reduction, and that as soon as it was removed no obstacle remained. I then naturally desired to know whether this dislocation ever occurs without the complication of the misplaced tendon; and if so, are such cases difficult of reduction? A little reflection on the conformation of these parts convinced me that it was not a necessary condition of the dislocation, but rather an accident depending on the force and direction of the violence that produced it. Again, I was also persuaded that it must be possible to diagnose this condition; for if the tendon remained *in situ* it would be perceptible, stretching over the end of the metacarpal bone, and drawn away from the first phalanx by the altered position of that bone. I had not long to wait for a reply to these inquiries, for my next case afforded all the information that I desired, and justified my anticipations.

Case 2.—A simple dislocation upwards and backwards of the first phalanx of the thumb. A careful examination soon after the accident occurred, and before any attempt at reduction had been made, enabled me to decide that the tendon was *not* displaced from its position between the tubercles on the lower end of the metacarpal bone, but could be recognized as a distinct band (especially when slight traction was made) passing from bone to bone. Then, as to the reduction, slight force only was required to restore the bones to their proper positions—viz: by simple extension from the last phalanx.

Having, then, this positive and negative evidence of the difficulty arising from the interposition of the tendon, I next sought for the best means of overcoming it. I conceived that the tendon might be carried back to its proper place by manipulation merely, without having recourse to division, and so leave the structures uninjured. I therefore devised the following procedure, and determined to test its application as soon as an opportunity permitted. The wrist being fully bent, so as to relax the long flexor tendon, let the surgeon take the thumb in one hand and abduct it from the fingers, while with the other hand he steadies the metacarpal bone; he then is to rotate the thumb so as to make the tendon retrace its course *forwards* and *inwards* around the lower end of the metacarpal bone, using the first phalanx as a lever in this intention. If this does not succeed, let him hyper-extend the first phalanx, so as to stretch the flexor tendon, rotate the phalanx *outwards*, and then carry it round the *inner* tubercle of the metacarpal bone, so as to dislodge the tendon from between the ends of the bones.

Case 3.—A simple dislocation of the first phalanx upon the dorsum of the metacarpal bone. No trace of the tendon could be discovered. Attempts to reduce the dislocation by extension had been made, and were renewed, that the manipulation might be fairly tested after other means had failed. The tendon was readily replaced by this means, co-aptation restored, and no tendency to displacement left.

I believe that I was thus enabled to place the argument on a basis so solid and satisfactory that it is impossible to resist its validity—indeed, that my cases reduced the matter to a demonstration. I will not dogmatise so far as to insist that all difficulty arises from the cause to which I was then induced to attribute it; for I can easily conceive that cases may be complicated by the altered positions of the flexor brevis as well as by the lateral ligaments. Still, I am convinced how important it is in all scientific inquiries to be well assured of our conclusions; that we may know how to apply our science with energy and decision; and that it is both politic and philosophical to be content with *one* solution of a problem so long as it enables us to act with effect; though it is equally right that we should remain open to conviction, however satisfied we may be with our opinions, when they are shown to be either controvertible or inadequate.

Since my attention has been directed to these cases, I have had much reason to believe that dislocation of the fingers at the metacarpo-phalangeal joints are also complicated by the malposition of their tendons; and, acting on this conviction, I have succeeded in reducing them by mere manipulation, after considerable force had been vainly applied.

I am induced to call the attention of the profession to this important subject from noticing that the valuable surgical works that have emanated from the press of late have not embodied or endorsed this view of the difficulties. The ligaments are still considered the principal obstacles to reduction by authors generally, though I feel assured that a more extensive observation of these cases by the great body of practical surgeons will confirm my own convictions, and lead to the adoption of a better and more successful mode of treatment.

